# Two Decades of CABG in the UK: A Propensity Matched Analysis of Outcomes by Conduit Choice

**DOI:** 10.3390/jcm13164717

**Published:** 2024-08-12

**Authors:** Georgia R. Layton, Shubhra Sinha, Massimo Caputo, Gianni D. Angelini, Daniel P. Fudulu, Mustafa Zakkar

**Affiliations:** 1 Department of Cardiovascular Sciences, University of Leicester, Leicester LE1 7RH, UK; 2 Department of Cardiac Surgery, University Hospitals of Leicester NHS Trust, Leicester LE1 5WW, UK; 3 Bristol Heart Institute, University of Bristol, Bristol BS8 1QU, UK

**Keywords:** ischaemic heart disease, coronary artery bypass grafting, CABG, saphenous vein, long saphenous vein, venous conduit, arterial conduit

## Abstract

**Background/Objectives**: Grafting of LIMA to LAD has long been considered the gold-standard conduit choice for patients undergoing CABG. Despite this, the LSV remains the most used conduit by volume and some patients may not receive even a single arterial conduit. However, the outcomes in this group are not frequently explored. This study, therefore, compares in-hospital outcomes of patients who underwent CABG without any arterial conduits to those who received at least one arterial conduit. **Methods**: Retrospective propensity-matched database analysis of consecutive patients undergoing CABG in the UK between 1996 and 2019 using data from the National Adult Cardiac Surgery Audit. **Results**: 335,144 patients underwent CABG, with 6% receiving venous conduits only; matched outcomes are reported for 39,812 patients. In both unmatched and matched groups, we found a significant increase in mortality with the use of veins only (matched mortality 5.3% vs. 3.8%, *p* < 0.001) with estimated treatment effect for mortality OR 1.43, *p* < 0.001 (95% CI: 1.31–1.57). We also identified greater rates of post-operative dialysis, IABP insertion, and length of hospital stay in this group. **Conclusions**: We identified a significant increase in in-hospital mortality with the use of veins only compared to using at least one arterial graft to the LAD. While a single arterial graft should be prioritised wherever possible, venous revascularisation retains a critical role for specific patients. We must, therefore, continue to conduct research addressing the mechanisms underlying and propagating vein graft disease in order better to optimise outcomes for this niche patient group after CABG.

## 1. Introduction

Ischaemic heart disease is commonly attributed to increased morbidity and mortality around the world [1]. Revascularisation of significantly diseased coronary arteries can be achieved using percutaneous coronary intervention (PCI) or coronary artery bypass graft surgery (CABG), depending upon the complexity and extent of coronary disease, as well as patient characteristics. Different conduits can be used during CABG, including the left internal mammary artery (LIMA), the long saphenous vein (LSV), and other arterial grafts, such as the radial artery (RA) and the right internal mammary artery (RIMA).

LIMA to left anterior descending artery (LAD) is considered the gold standard for all patients undergoing CABG (when feasible) due to its excellent long-term patency and improved long-term outcomes [2,3,4]. However, there are occasions when the LIMA cannot be used, and alternative arterial conduits, such as RIMA, may be used in its place [5,6]. Despite this, some patients undergoing CABG will still receive grafts with only the long saphenous vein (LSV). Although the LSV remains the most used conduit by number of grafts, it is associated with high rates of mid- to long-term stenosis or complete occlusion due to the development of intimal hyperplasia or vein graft disease (VGD). VGD significantly reduces long term patency of conduits [7,8], even if used to graft the LAD [2,9]. Reviewing the national data series of CABG in the United Kingdom (UK), we identified the cohort of patients who underwent CABG utilising veins only, even to the LAD territory (TV-CABG), and our objective in this study is to compare the outcomes in this cohort, contrasted to those who received at least one arterial conduit, including an arterial conduit to the LAD (SD-CABG), using both unmatched and propensity-matched analysis.

## 2. Materials and Methods

### 2.1. Participants

**Figure 1:**
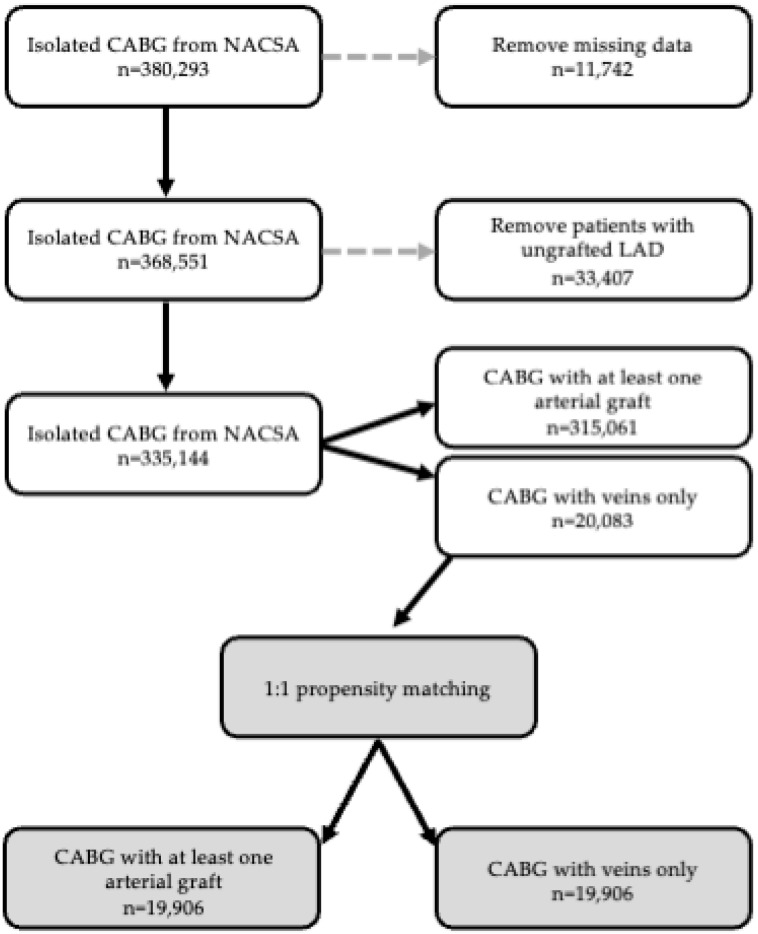
Consort diagram of data flow and excluded patient populations.

Data for all patients who underwent isolated CABG in the UK between February 1996 and March 2019 were included. Patients who underwent CABG without grafting to their LAD or patients receiving concomitant non-CABG intervention (i.e., valve replacement), paediatric and adult congenital procedures, transplants, mechanical support device insertion, or patients whose operative definitions were missing were excluded (Figure 1). Data were collected regarding pre-operative patient exposures and clinical status, procedural factors, and postoperative outcomes during the index hospital admission.

The National Adult Cardiac Surgery Audit (NACSA) began collecting prospective data from all major cardiac surgery procedures in the UK NHS in 1996. Data are collected pertaining to demographic and key peri-operative clinical information, as well as major in-hospital outcomes, including stroke, myocardial infarction, and death. The database structure from input to analysis has been described previously [10], as have definitions and variables [11]. Following local entry and initial validation, data are uploaded to the National Institute for Cardiovascular Outcomes Research (NICOR) via a web portal. Central validation is performed using logical rules, and missing data reports are generated for all primary variables (e.g., Euroscore II risk prediction, patient identifiers, and key outcome data). Missing data are identified at this point, and the overall percentage of missing data for baseline information within the database was reported in the range of 1.7% [10]. Missing data for the current study were handled by exclusion and reported in the baseline and outcome tables.

Missing or conflicting data entries regarding in-hospital mortality are backfilled and validated through linking with Office for National Statistics (ONS) census database. Following validation, data are forwarded for data cleaning by a healthcare informatics team. Any duplications within the records are removed and any discrepancies in the transcription are corrected, followed by resolution of any clinical and temporal conflicts.

### 2.2. Ethics

As this study utilised retrospective analysis of the NACSA from the NICOR database, individual patient consent for data usage was not required. This study is part of a Health Research Authority and Health and Care Research Wales-approved project, was registered with the Integrated Research Application System (project ID: 257758), a waiver for patient consent was obtained and was conducted strictly in line with ethical standards specified by the Declaration of Helsinki (1964) [12] and its later amendments, as well as General Data Protection Regulations. The selected end-points included in-hospital mortality defined as death by any cause that occurred at any time before discharge from index hospitalization, regardless the length of hospital stay, the need for new post-operative renal replacement therapy, cerebrovascular accidents defined as any new postoperative stroke or transient ischaemic attack (TIA) confirmed clinically or by computed tomography scan, severe low cardiac output (LCO), requiring intraoperative or postoperative intra-aortic balloon pump (IABP), return back to theatre, and length of hospital stay.

### 2.3. Statistical Analysis

Categorical variables were summarised as counts and percentages and compared using a Pearson’s Chi-squared test or Fisher’s exact test. We used the Shapiro–Wilks test to assess the normality of the distribution of continuous data. Our continuous data were non-normally distributed and were summarised as a median with interquartile range (IQR) and analysed using the Wilcoxon rank sum test.

Propensity score matching was performed using 1:1 nearest neighbour matching without replacement using the baseline characteristics from the NACSA dataset, which we determined to be best related to clinical outcome including: age, sex, body mass index (BMI), pulmonary disease, peripheral vascular disease (PVD), poor mobility, renal impairment defined as creatinine more than 200 μmol/L, unstable angina defined as Canadian Cardiovascular Society (CCS) Angina Scale of 4, severe breathlessness defined as New York Heart Association (NYHA) score of 4, moderate, poor, or very-poor left ventricular function, insulin dependent diabetes, previous cardiac surgery, critical peri-operative state, recent myocardial infarction (MI), pulmonary hypertension, urgent, emergency or salvage surgery urgency, use of mechanical support, and use of cardiopulmonary bypass (CPB).

After matching, all standardised mean differences (SMD) for the covariates were checked using love plots and the adequate balance was set to be below 0.1 (Supplementary Figure S1). All SMDs were below 0.1. Matched variables were compared using a Wilcoxon rank sum paired test for continuous data and a McNemar test for binary paired data [13].

To estimate the effect of CABG using vein only versus using at least one arterial graft, we fitted a logistic regression model with mortality as a binary outcome and the use of vein only and the matching covariates as predictors (double adjustment). We included the full matching weights in the estimation. The “glm ()” function was used to fit the outcome, and the comparisons() function in the “marginal effects” package was used to perform g-computation in the matched sample to estimate the average treatment effect in patients undergoing CABG using veins-only. A cluster-robust inference with matching stratum membership as the clustering variable was used to calculate the marginal OR and confidence intervals [14,15,16].

Significance was set at a *p* value < 0.05. Analyses were performed using R (Version 4.1.1) [17] and R Studio (Version 1.4.1103) [18] using packages tidyverse, Matchit, marginal effects, ggplot2, and flextable. The data flow diagram was created using Inkscape 1.3.2 for macOS and the rest of the figures and tables using gtsummary.

## 3. Results

### 3.1. Unmatched Population Cohort

**Figure 2:**
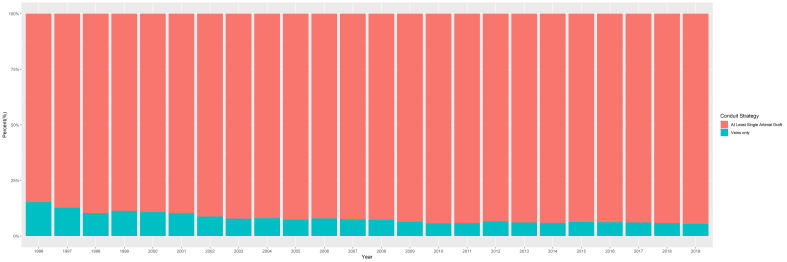
The percentage of all annual CABG cases using venous-only conduits (green) versus at least one arterial conduit (orange) over time between 1996 and 2019.

In total, 335,144 patients who underwent CABG were included in this study. The rates of CABG procedures using venous only conduits during the time frame of this analysis are depicted in Figure 2 and remained fairly constant throughout the study period.

#### 3.1.1. Baseline and Intra-Operative Patient Characteristics

In total, 335,144 patients were included in this study. Of these, 94% (315,061) had at least one arterial graft to LAD and may have had additional arterial grafts to other vessels (SD-CABG). Comparatively, 6% (20,083) of patients received only venous conduits in any territory (TV-CABG). Baseline patient and operative characteristics of the overall and unmatched populations are summarised in Table 1.

Details of arterial conduit use in the SD-CABG group are reported in Supplementary Table S1. Within this cohort, almost all patients had LIMA either as a pedicle (97%) or free graft (1.6%), with other conduits being used significantly less for both single- or multi-arterial grafting approaches.

The overall median age of the whole study population was 49 years (IQR 42–55), with patients in the TV-CABG cohort being significantly older than SD-CABG patients (53 (IQR 46–59) vs. 48 (IQR 41–55), respectively, *p* < 0.001). Overall, 19% (62,898) of patients were female, and there were significantly more women in the TV-CABG cohort compared to SD-CABG (30%, vs. 18%, *p* < 0.001). Median BMI was 27.8 (IQR 25.4–30.9), which was lower in the TV-CABG cohort compared to SD-CABG (27.3, IQR 24.6–30.3) vs. median 27.8, IQR 25.4–30.9), respectively, *p* < 0.001). Chronic pulmonary disease was present in 11% (38,300) of patients overall and was more common in the TV-CABG group compared to the SD-CABG group (17% vs. 11%, respectively, *p* < 0.001). Similarly, PVD occurred in 13% (42,983) overall and was more frequent in the TV-CABG group compared to SD-CABG group (12% vs. 19%, respectively, *p* < 0.001).

Overall, 64% (213,994) of all cases were elective, with more elective cases in the SD-CABG compared to TV-CABG (65% vs. 50%, respectively, *p* < 0.001). Of the 2.1% (7161) emergency cases, 8.7% (1744) had veins only compared to 1.7% (5417) in the SD-CABG group (*p* < 0.001). Similarly, more cases in the salvage patients were in the TV-CABG group compared to the SD-CABG group (1.9% vs. <0.1%, respectively, *p* < 0.001). Critical preoperative status was present in 2.23% overall and was more common in the TV-CABG cohort compared to SD-CABG (8.5% vs. 1.9%, respectively, *p* < 0.001). More patients in the TV-CABG group had poor or very-poor LV function compared to the SD-CABG group (2.7 vs. 1.5%, *p* < 0.001 and 1% vs. 0.4%, *p* < 0.001, respectively). IABP was utilised prior to surgery in 2.7% (2653) patients overall and was more frequently used in the TV-CABG cohort compared to SD-CABG cohort (9.45 vs. 2.4, respectively, *p* < 0.001), consistent with the higher rates of urgent and emergent surgery in the TV-CABG group. The overall median EuroScore II was 1.1 (IQR 0.75–1.64), with the TV-CABG group having higher EuroScore II (1.66 (IQR 1–3) vs. 1 (IQR 0.7–1.58), respectively, *p* < 0.001).

In total, 85% of all procedures (284,343) were performed using CPB, with a median overall CPB time of 78 min (IQR 58–99); this was less in the SD-CABG cohort, with a median time of 78 min (IQR 58–98) compared to TV-CABG group’s 80 min (IQR 61–104), *p* < 0.001. The overall cross-clamp time for on-pump cases was 44 min (IQR 31–59) and was less in patients receiving TV-CABG (38 min, IQR 27–54) compared to SD-CABG group (44 min, IQR 31–59) (*p* < 0.001).

#### 3.1.2. In-Hospital Outcomes in the Whole Population

In-hospital outcomes for the overall population and unmatched groups are reported in Table 2. All-cause in-hospital mortality was 1.8% (5847), which was significantly higher in the TV-CABG group compared to SD-CABG (5.7% vs. 1.5%, respectively, *p* < 0.001). Postoperative CVA occurred in 1.7% (5447) of patients overall and was more frequent in the TV-CABG group (2.6% vs. 1.7%, respectively, *p* < 0.001). New post-operative hemofiltration or dialysis occurred in 2.1% (6330) and was more common in the TV-CABG cohort compared to SD-CABG (4.4% vs. 2%, respectively, *p* < 0.001).

Significant postoperative bleeding requiring re-exploration occurred in 2.9% (9821) and was more frequent in the TV-CABG cohort compared to SD-CABG (4% vs. 2.9%, respectively, *p* < 0.001). Overall, 1% (817) had postoperative deep sternal wound infection (DSWI), which was slightly higher in the TV-CABG cohort (1.5% vs. 1%, *p* < 0.004) but we note there was a very high rate of data missingness for post-operative infection specifically and therefore these data were excluded.

### 3.2. Matched Population Cohort

Following matching, 39,812 patients were included in the analysis (19,906 per group). The patient and operative baseline characteristics and the associated SMD between groups after matching are reported in Table 3 and Supplementary Figure S1. Post matching, LIMA remained the most commonly used graft, either as a pedicle (96%) or free graft (1.7%), in the SD-CABG group (Supplementary Table S1). Pre-matching SMD are reported in Table 1.

#### 3.2.1. Baseline and Intra-Operative Patient Characteristics

Post-matching, there was no residual imbalance in the matching variables—all SMDs were below 0.05 (Table 1 and Table 3).

#### 3.2.2. In-Hospital Outcomes

In-hospital outcomes for the matched population are presented in Table 4. All cause in-hospital mortality remained significantly higher in the TV-CABG group compared to the matched SD-CABG group (5.3%% vs. 3.8%, respectively, *p* < 0.001). More patients in the TV-CABG group required intra/postoperative IABP insertion compared to SD-CABG (8.4% vs. 5.4%, respectively, *p* < 0.001). Additionally, more patients in the TV-CABG group required new post-operative renal replacement therapy compared to SD-CABG (4.3% vs. 3.6%, respectively, *p* = 0.002). Rates of both TIA and CVA were similar between both graft strategy groups (*p* = 0.5 and *p* = 0.072, respectively). Other postoperative complications, including re-exploration for bleeding or graft complications, were similar between groups following matching. When estimating the treatment effect of using vein only versus at least one arterial graft, we found a significant increase in mortality with the use of solely venous conduits when compared to using at least one arterial graft (marginal OR 1.43, 95% C.I: 1.31–1.57, *p* < 0.001). Within the matched groups, CPB time and cross-clamp time, which were not used as matching variables, were similar to those between the unmatched sub-groups; CPB time was a median of 80 min in both groups, but cross-clamp time was prolonged in the SD-CABG group at 45 min (IQR 33–59) compared to 38 min (IQR 27–54) in the TV-CABG group (*p* < 0.001).

## 4. Discussion

This is the largest published analysis of patient outcomes following isolated CABG procedures comparing patients based on the use of veins only for revascularisation (TV-CABG) to at least a single arterial graft to the LAD (SD-CABG) in the UK. In the unmatched analysis, 335,144 patients were included. Of those, 94% (315,061) of patients received an artery to at-least the LAD while 6% (20,083) of patients received total venous revascularisation. Baseline characteristics and risk factors associated with increased adverse outcomes were seen more frequently in the cohort of patients that underwent veins only CABG. More patients in the TV-CABG group had poor or very poor LV function or underwent non-elective surgery compared to SD-CABG group. Thus, the median EuroScore II risk was significantly higher in the TV-CABG group. Expectedly, IABP use before surgery was utilised more frequently in the TV-CABG cohort compared to SD-CABG cohort. All-cause in-hospital mortality was significantly higher in the TV-CABG group compared to SD-CABG (5.7% vs. 1.5%, *p* < 0.001). Intra/post operative IABP insertion was required more often in the TV-CABG group when compared to SD-CABG. Post operative CVA occurred more frequently in the TV-CABG group, and more patients in the TV-CABG group required new post-operative hemofiltration or dialysis. Considering these major differences in preoperative characteristics between the two conduit strategy groups, we carried out propensity matching. Following matching, 39,812 patients were included in the analysis (19,906 per group).

Post-matching, there were no major differences in the baseline characteristics. Interestingly, fewer patients in the TV-CABG had urgent surgery compared to SD-CABG and more in the TV-CABG had pre-operative IABP. All cause in-hospital mortality remained significantly higher in the TV-CABG group compared to the matched SD-CABG group (5.3%% vs. 3.7%, *p* < 0.001). When estimating treatment effect, we found a significant increase in mortality with the use of solely venous conduits during CABG compared to using at least one arterial graft (OR 1.43, 95% C.I: 1.31–1.57, *p* < 0.001). Furthermore, more patients in the TV-CABG required intra/postoperative IABP insertion compared to SD-CABG; in addition, a higher number of patients in the TV-CABG group required new post-operative renal replacement therapy compared to SD-CABG.

There is a well-established benefit from revascularisation of the LAD territory, particularly with LIMA, and published evidence suggests that loss of this benefit may render the outcomes of CABG similar to those of other treatments, including PCI [19]. Thus, LIMA anastomosed to the LAD remains the gold standard for patients undergoing surgical revascularisation and an overwhelming proportion of CABG patients will receive this. However, the LIMA may not be harvested or may be of inadequate quality for use for several different reasons [20,21]. Under these circumstances, an alternative arterial conduit, such as RIMA or RA, can be considered. The use of RIMA is associated with good outcomes post-surgery [5,6], Previous report, however, suggested that the use of RA to LAD can be an independent predictor of increased MACE after RA-CABG [22], thus its use to LAD should be treated with caution and requires more investigation. However, technical factors, such as site or severity of coronary stenosis or a clinical reason to avoid harvest of an additional arterial conduit, may render veins the only alternative conduit option. Additionally, some surgeons will elect to use veins as a primary conduit of choice in an unstable patient requiring emergency revascularisation [23,24,25]. Although in our series we cannot identify the reasons for not using the LAD or another arterial grafts, our matching analysis suggest that even in cases of emergency surgery, the use of arterial grafts remains associated with better survival. One observation from the matched population is that salvage surgery still frequently utilised a TV strategy, with 1.1% (n = 304) of the TV group undergoing salvage surgery compared to only 0.7% (n = 183) in the AG group (*p* < 0.001) despite similar rates of elective, urgent, and emergency surgery between cohorts. This elevated burden of salvage surgery suggests that many patients may have received TV in line with surgeon preference given the dire circumstances during salvage situations considering the relative ease of harvesting venous conduits [26].

Our results confirm that the use of at least one arterial graft to the LAD territory is favourable in patients undergoing CABG, and these findings are in line with contemporary outcome data, even accepting that when LSV is grafted into LAD, patency may be better than other territories [2,9]. However, the outcomes of TV grafting remain inferior to arterial grafts [9,27]. Interestingly, previous analysis of the same database presented here identified that the improved outcomes do not persist beyond single arterial grafting with the use of MAG [3]. This suggest that the placement of an arterial graft to LAD seems to be the most valuable part of CABG and every attempt should be utilised to use it. It is important to recognise that there will always be a small cohort of patients where arterial grafts cannot be used despite every effort. This group of patients are subject to higher rates of adverse outcomes and greater mortality after surgery. This places a spotlight on the importance of continuing research to understand the mechanisms underpinning the onset of vein graft disease, focusing on acute changes that occur within the vein immediately after harvest and implantation to improve its patency rates alongside optimised preservation solutions and post-surgery medical therapy [7,8].

### Limitations

This study is a retrospective analysis of data collected over more than twenty years. During this time, there were significant changes in technique and provision of resources for cardiac surgery. There is no evidence to suggest there have been any interventions widely implemented in UK practice that would introduce significant bias upon the outcomes of one conduit group over the other. However, we acknowledge that this broad timespan, as well as its retrospective nature, is a limitation of performing analysis of databases such as the NACSA. Similarly, data within NACSA pertain only to in-hospital outcomes and so it is not possible to assess medium- or long-term outcomes in this study. Overall, our data had reporting rates of more than 99%. These data are validated and submitted nationally by all operating units in the UK and are used for annual public reporting on outcomes after cardiac surgery. Therefore, although retrospective, its consistency and accuracy are well documented.

Although these study groups are well-matched, there may be residual confounding from factors not recorded within the database. These may include, but are not limited to, inter group variation of incomplete revascularisation, pharmacological compliance, and surgeon experience or case volume. NACSA compiles data from several hundred surgeons across all units in the UK and so, in combination with the large population size, statistical averaging suggests any bias introduced by any surgeon- or unit-based factor is likely to have an insignificant impact on our findings. Some critical data, such as the complexity of coronary disease patterns, vessel quality, and distribution of calcification, cannot be evaluated within the NACSA data. It is, therefore, not possible to exclude correlation between more challenging disease distributions and conduit choice, which in turn may have contributed to worse in-hospital outcomes in the total venous revascularisation group. The lack of long-term data compounds our inability to assess the impact of incomplete revascularisation or differences in graft patency. However, given the well-established body of evidence showing improved arterial conduit patency compared to venous, is it likely this would identify a much greater mortality burden in the total venous revascularisation group.

## 5. Conclusions

We identified a significant excess in mortality with the use of veins only compared to CABG performed using at least one arterial graft to the LAD. While the use of a single arterial graft should be prioritised for all patients wherever possible, total venous revascularisation retains a critical role for a specific subset of patients, particularly for emergency presentations or when arterial conduit has been planned but is not useable for any reason. We must, therefore, continue to conduct research addressing the mechanisms underlying and propagating vein graft disease to better optimise outcomes for this niche patient group after CABG.

## Figures and Tables

**Table 1:** Baseline and operative characteristics and standardized mean differences prior to propensity matching in the whole cohort.

		Conduit Strategy			
Characteristic	Overall, N = 335,144 ^1^	At Least Single Arterial Graft, N = 315,061 ^1^	Veins Only, N = 20,083 ^1^	*p*-Value ^2^	Standardised Mean Difference
**Age (years)**	49 (42, 55)	48 (41, 55)	53 (46, 59)	<0.001	0.429
**Gender**				<0.001	0.262
Female	62,898 (19%)	56,857 (18%)	6041 (30%)		
**Pulmonary Disease**				<0.001	0.156
	38,300 (11%)	34,896 (11%)	3404 (17%)		
**Peripheral Vascular Disease**				<0.001	0.166
	42,983 (13%)	39,177 (12%)	3806 (19%)		
**Poor mobility pre-op**				<0.001	0.068
	7228 (2.2%)	6563 (2.1%)	665 (3.3%)		
**Creatinine > 200 μmol/L**				<0.001	0.118
	5651 (1.7%)	4885 (1.6%)	766 (3.8%)		
**Unstable angina**				<0.001	0.296
	59,649 (18%)	53,491 (17%)	6158 (31%)		
**Good LV Function**	289,028 (86%)	272,139 (86%)	16,889 (84%)	<0.001	
**Moderate LV Function**				0.016	0.173
	39,301 (12%)	36,839 (12%)	2462 (12%)		
**Poor LV Function**				<0.001	0.072
	5266 (1.6%)	4730 (1.5%)	536 (2.7%)		
**Very poor LV Function**				<0.001	0.056
	1577 (0.5%)	1377 (0.4%)	200 (1.0%)		
**Redo**				<0.001	0.257
	6472 (1.9%)	4711 (1.5%)	1761 (8.8%)		
**Critical Pre-operative State**				<0.001	0.237
	7724 (2.3%)	6010 (1.9%)	1714 (8.5%)		
**Recent MI**				<0.001	0.131
	83,027 (25%)	76,908 (24%)	6119 (30%)		
**Pulmonary Hypertension**				<0.001	0.051
	1684 (0.5%)	1488 (0.5%)	196 (1.0%)		
**Elective case**	213,994 (64%)	203,893 (65%)	10,101 (50%)	<0.001	
**Urgent case**				<0.001	0.113
	113,138 (34%)	105,310 (33%)	7828 (39%)		
**Emergency case**				<0.001	0.247
	7161 (2.1%)	5417 (1.7%)	1744 (8.7%)		
**Salvage case**				<0.001	0.133
	555 (0.2%)	181 (<0.1%)	374 (1.9%)		
**NYHA I**				<0.001	-
	103,164 (31%)	97,973 (31%)	5191 (26%)		
**NYHA II**				<0.001	-
	152,847 (46%)	144,602 (46%)	8245 (41%)		
**NYHA III**				<0.001	-
	68,942 (21%)	63,755 (20%)	5187 (26%)		
**NYHA IV**				<0.001	0.173
	10,191 (3.0%)	8731 (2.8%)	1460 (7.3%)		
**CCS 0**				<0.001	-
	33,417 (10.0%)	31,578 (10%)	1839 (9.2%)		
**CCS 1**				<0.001	-
	29,928 (8.9%)	28,514 (9.1%)	1414 (7.0%)		
**CCS 2**				<0.001	-
	117,837 (35%)	112,404 (36%)	5433 (27%)		
**CCS 3**				0.006	-
	105,607 (32%)	99,103 (31%)	6504 (32%)		
**CCS 4**				<0.001	0.246
	48,355 (14%)	43,462 (14%)	4893 (24%)		
**BMI**	27.8 (25.4, 30.9)	27.8 (25.4, 30.9)	27.3 (24.6, 30.3)	<0.001	0.150
**Mechanical Support**				<0.001	0.105
Required	2679 (0.8%)	2220 (0.7%)	459 (2.3%)		
**Pump Case**				<0.001	0.344
Off pump	50,801 (15%)	49,409 (16%)	1392 (6.9%)		
On pump	284,343 (85%)	265,652 (84%)	18,691 (93%)		
**Diet controlled Diabetes**	13,830 (4.1%)	12,754 (4.0%)	1076 (5.4%)	<0.001	-
**Diabetes on oral medications**	46,153 (14%)	43,403 (14%)	2750 (14%)	0.7	-
**Diabetes on insulin**				<0.001	0.029
	25,338 (7.6%)	23,664 (7.5%)	1674 (8.3%)		
**EuroScore II**	1.05 (0.75, 1.64)	1.02 (0.74, 1.58)	1.66 (1.03, 2.99)	<0.001	-
**CPB Time(mins)**	78 (58, 99)	78 (58, 98)	80 (61, 104)	<0.001	-
Missing	36,256	34,657	1599		
**Cross Clamp Time(mins)**	44 (31, 59)	44 (31, 59)	38 (27, 54)	<0.001	-
Missing	36,594	34,943	1651		
**Preop IABP**				<0.001	-
Used	2653 (0.8%)	2200 (0.7%)	453 (2.3%)		

^1^ Median (IQR) or Frequency (%). ^2^ Wilcoxon rank sum test; Pearson’s Chi-squared test; Fisher’s exact test.

**Table 2:** In-hospital outcomes prior to propensity matching in all patients.

Conduit Strategy				
Characteristic	Overall, N = 335,144 ^1^	At Least Single Arterial Graft, N = 315,061 ^1^	Veins Only, N = 20,083 ^1^	*p*-Value ^2^
**Mortality**	5847 (1.8%)	4711 (1.5%)	1136 (5.7%)	<0.001
Missing	2007	1910	97	
**CVA**				<0.001
	5447 (1.7%)	4973 (1.7%)	474 (2.6%)	
Missing	21,617	19,963	1654	
**TIA**				<0.001
	11,246 (3.6%)	10,326 (3.5%)	920 (5.0%)	
Missing	21,617	19,963	1654	
**Dialysis**	6330 (2.1%)	5555 (2.0%)	775 (4.4%)	<0.001
Missing	38,497	35,888	2609	
**Intraoperative/postoperative IABP insertion**				<0.001
Used	2687 (0.8%)	2272 (0.7%)	415 (2.1%)	
**Return to theatre for bleeding/tamponade**	9821 (2.9%)	9022 (2.9%)	799 (4.0%)	<0.001
**Return to theatre for graft complications**	425 (0.1%)	391 (0.1%)	34 (0.2%)	0.081
**Return to theatre for rhythm issues**	34 (<0.1%)	34 (<0.1%)	0 (0%)	0.3
**Pre-op Length of Stay (days)**	1.0 (1.0, 2.0)	1.0 (1.0, 2.0)	1.0 (1.0, 4.0)	<0.001
Missing	7038	6507	531	
**Post-op Length of Stay (days)**	6.0 (5.0, 9.0)	6.0 (5.0, 8.0)	7.0 (6.0, 11.0)	<0.001
Missing	5869	5379	490	
**Total Length of Stay(days)**	8 (7, 13)	8 (7, 12)	10 (7, 17)	<0.001
Missing	9566	8777	789	

^1^ Median (IQR) or Frequency (%). ^2^ Wilcoxon rank sum test; Pearson’s Chi-squared test; Fisher’s exact test.

**Table 3:** Baseline patient and operative characteristics and the associated standardised mean differences following propensity matching.

Characteristic	After Matching		
	At Least Single Arterial Graft, N = 19,906 ^1^	Veins Only, N = 19,906 ^1^	Standardised Mean Difference
**Age (years)**	53 (47, 58)	53 (46, 59)	0.023
**Gender**	6122 (31%)	5966 (30%)	0.017
**Pulmonary Disease**	3500 (18%)	3374 (17%)	0.016
**Peripheral Vascular Disease**	3800 (19%)	3763 (19%)	0.004
**Poor mobility pre-op**	693 (3.5%)	657 (3.3%)	0.010
**Creatinine > 200 μmol/L**	757 (3.8%)	739 (3.7%)	0.004
**Unstable angina**	6108 (31%)	6021 (30%)	0.009
**Moderate LV Function**	2433 (12%)	2447 (12%)	0.002
**Poor LV Function**	540 (2.7%)	525 (2.6%)	0.004
**Very poor LV Function**	187 (0.9%)	187 (0.9%)	0.000
**Diabetes on insulin**	1659 (8.3%)	1659 (8.3%)	0.000
**Redo**	1673 (8.4%)	1698 (8.5%)	0.004
**Critical Pre-operative State**	1417 (7.1%)	1557 (7.8%)	0.025
**Recent MI**	5947 (30%)	6018 (30%)	0.007
**Pulmonary Hypertension**	217 (1.1%)	191 (1.0%)	0.013
**Preoperative IABP**	415 (2.1%)	437 (2.2%)	0.007
**Urgent case**	8046 (40%)	7821 (39%)	0.023
**Emergency case**	1659 (8.3%)	1697 (8.5%)	0.006
**Salvage case**	150 (0.8%)	251 (1.3%)	0.037
**NYHA IV**	1405 (7.1%)	1393 (7.0%)	0.002
**CCS 4**	27.3 (24.7, 30.2)	27.3 (24.6, 30.3)	0.008
**BMI**	27.3 (24.7, 30.2)	27.3 (24.6, 30.3)	0.004
**Mechanical Support**	421 (2.1%)	443 (2.2%)	0.004
**Pump Case**	18,524 (93%)	18,516 (93%)	0.001

^1^ Median (IQR) or Frequency (%).

**Table 4:** In-hospital outcomes following propensity matching.

	Conduit Strategy		
Characteristic	At Least Single Arterial Graft, N = 19,906 ^1^	Veins Only, N = 19,906 ^1^	*p*-Value ^2^
**CPB Time(mins) ^3^**	80 (61, 100)	80 (61, 104)	<0.001
Missing	1261	1594	
**Cross Clamp Time(mins) ^3^**	45 (33, 59)	38 (27, 54)	<0.001
Missing	1314	1634	
**Mortality**			<0.001
	746 (3.8%)	1054 (5.3%)	
Missing	127	96	
**CVA**			0.072
	437 (2.4%)	473 (2.6%)	
Missing	1512	1639	
**TIA**			0.5
	890 (4.8%)	911 (5.0%)	
Missing	1512	1639	
**Dialysis**			0.042
	653 (3.8%)	749 (4.3%)	
Missing	2544	2574	
**Intraoperative/postoperative IABP insertion**			<0.001
Used	330 (5.5%)	402 (8.4%)	
Missing	13,905	15,104	
**Return to theatre for bleeding/tamponade**			0.2
Yes	733 (3.7%)	779 (3.9%)	
**Return to theatre for graft complications**			0.8
Yes	35 (0.2%)	32 (0.2%)	
**Return to theatre for rhythm issues**			
Yes	4 (<0.1%)	0 (0%)	
**Pre-op Length of Stay (days)**	1.0 (1.0, 4.0)	1.0 (1.0, 4.0)	0.8
Missing	416	529	
**Post-op Length of Stay (days)**	7 (6, 10)	7 (6, 11)	0.002
Missing	336	488	
**Total Length of Stay(days)**	9 (7, 16)	10 (7, 17)	<0.001
Missing	566	786	

^1^ Median (IQR) or Frequency (%). ^2^ Wilcoxon rank sum test; McNemar’s Chi-squared test with continuity correction; McNemar’s Chi-squared test. ^3^ Characteristic not used for propensity matching.

## Data Availability

The data underlying this article were provided by National Institute for Cardiovascular Outcomes Research by permission. Data will be shared on request to the corresponding author; with permission of the National Institute for Cardiovascular Outcomes Research.

## Supplementary Materials

The following supporting information can be downloaded at: https://www.mdpi.com/article/10.3390/jcm13164717/s1, Table S1: Types of arterial conduit used in the SD-CABG group for procedures utilising at least one arterial conduit, stratified by pre- and post-matching populations. Figure S1: Love plot showing differences of standardised mean between total venous and arterial conduit groups. The plot demonstrates no significant differences between these groups following propensity matching, suggesting good distributional balance of all listed baseline covariates in the matched samples. Whilst there is no clear consensus, a standardised difference of 0.1 is generally accepted to denote meaningful imbalance in a covariate [28].
