# Supplementary material for: Two Decades of CABG in the UK: A Propensity Matched Analysis of Outcomes by Conduit Choice
Source: J Clin Med. 2024 Aug 12;13(16):4717. doi: 10.3390/jcm13164717 (PMC11355931; doi:10.3390/jcm13164717)

<u>Supplementary Tables</u>

Supplementary Table S1: Types of arterial conduit used in the SD-CABG group, stratified by pre-and post-matching cohorts. All patients in this cohort received at least one arterial conduit.

**Supplementary Table S1 - Arterial grafts before matching**		
**Characteristic**	**Before matching**	**After matching**
	N = 315,061[1]	N = 19,906[1]
**Pedicled LIMA**	306,379 (97%)	19,149 (96%)
**Radial**	37,604 (12%)	1,894 (9.5%)
**Pedicled RIMA**	13,573 (4.3%)	625 (3.1%)
**Free LIMA**	4,967 (1.6%)	345 (1.7%)
**Free RIMA**	5,199 (1.7%)	229 (1.2%)
[1] Median (IQR) or Frequency (%)		

Supplementary Figure S1: Love plot showing differences of standardised mean between total venous and arterial conduit groups. The plot demonstrates no significant differences between these groups following propensity matching, suggesting good distributional balance of all listed baseline covariates in the matched samples. While there is no clear consensus, a standardised difference of 0.1 is generally accepted to denote meaningful imbalance in a covariate [29].

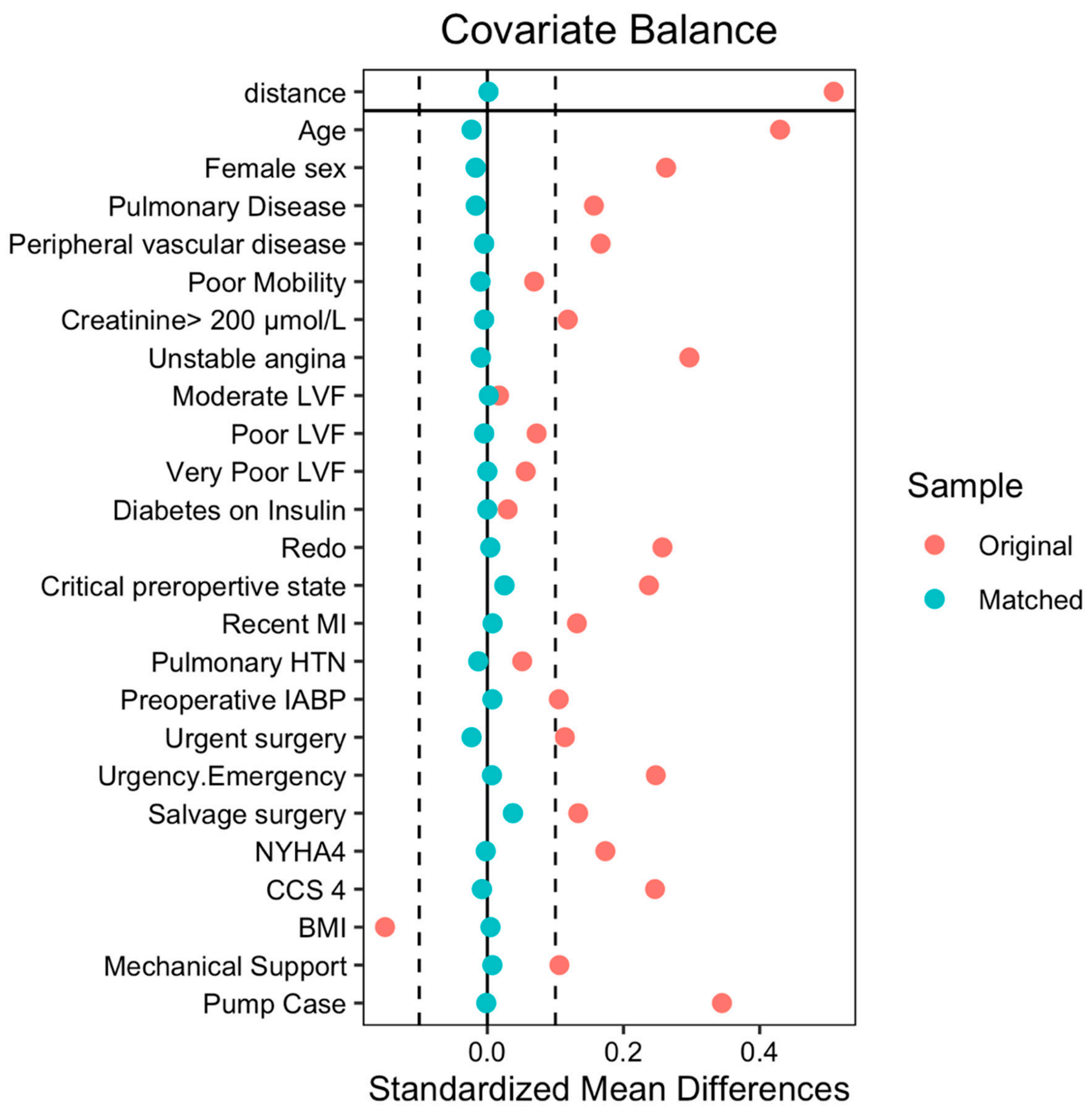

Supplement: Supplementary file 1 [file jcm-13-04717-s001.zip › jcm-3085211-supplementary.pdf]
